# Phenotypic and Genomic Properties of a Novel Deep-Lineage Haloalkaliphilic Member of the Phylum *Balneolaeota* From Soda Lakes Possessing Na^+^-Translocating Proteorhodopsin

**DOI:** 10.3389/fmicb.2018.02672

**Published:** 2018-11-13

**Authors:** Dimitry Y. Sorokin, Maria S. Muntyan, Stepan V. Toshchakov, Aleksei Korzhenkov, Ilya V. Kublanov

**Affiliations:** ^1^Winogradsky Institute of Microbiology, Research Center of Biotechnology of the Russian Academy of Sciences, Moscow, Russia; ^2^Department of Biotechnology, Delft University of Technology, Delft, Netherlands; ^3^Belozersky Institute of Physico-Chemical Biology, Lomonosov Moscow State University, Moscow, Russia; ^4^Immanuel Kant Baltic Federal University, Kaliningrad, Russia

**Keywords:** soda lakes, haloalkaliphilic, proteolytic, *Balneolaeota*, Na^+^-proteorhodopsin

## Abstract

Stable development of a heterotrophic bacterial satellite with a peculiar cell morphology has been observed in several enrichment cultures of haloalkaliphilic benthic filamentous cyanobacteria from a hypersaline soda lake in Kulunda Steppe (Altai, Russia). The organism was isolated in pure culture (strain Omega) using sonicated cyanobacterial cells as substrate and it was identified as a deep phylogenetic lineage within the recently proposed phylum *Balneolaeota*. It is an obligately aerobic heterotroph utilizing proteins and peptides for growth. The cell morphology significantly varied from semicircles to long filaments depending on the growth conditions. The cultures are red-orange colored due to a presence of carotenoids. The isolate is an obligate alkaliphile with a pH range for growth from 8.5 to 10.5 (optimum at 9.5–10) and moderately salt-tolerant with a range from 0.3 to 3 M total Na^+^ (optimum at 1 M). The genome analysis of strain Omega demonstrated a presence of gene, encoding a proteorhodopsin forming a separate branch in the sodium-translocating proteorhodopsin family. Experiments with washed cells of Omega confirmed light-dependent sodium export. A possible physiological role of the sodium proteorhodopsin in strain Omega is discussed. Phylogenomic analysis demostrated that strain Omega forms an deep, independent branch of a new genus and family level within a recently established phylum *Balneolaeota*.

## Introduction

Soda lakes are a specific variety of salt lakes which brines are dominated by sodium carbonates resulting in molar alkalinity in solution and a stable pH around 10. Despite double extreme conditions of high salt-high pH, soda lakes, in general, are highly productive (Melack, 1981, 1988; Kompantseva et al., 2009) harboring diverse haloalkaliphilic communities dominated by prokaryotes responsible for the cycling of important biogenic elements, such as carbon, nitrogen and sulfur (last reviewed: Antony et al., 2013; Sorokin et al., 2014, 2015; Sorokin, 2017). Thus, during the last 30 years, the soda lake prokaryotes have been a subject of intense fundamental as well as biotechnological research, as a limits-of-life case and a source of salt-tolerant and alkali-stable exo-enzymes, respectively (last reviewed: Zhao et al., 2014; Amoozegar et al., 2017).

Haloalkaliphilic oxygenic cyanobacteria are the dominant primary producers in soda lakes. The evidences on their diversity are not abundant, however. Moderately saline tropical soda lakes in Africa are dominated by *Spirulina*-*Cyanospira-Arthrospira* clade (Krienitz et al., 2013; Krienitz and Schagerl, 2016), while in Siberian moderate and hypersaline soda lakes, at severe continental climate, two benthic filamentous forms belonging to the genera *Nodosilinea* and *Geitlerinema* have been consistently detected (Samylina et al., 2014). In contrast to algae, the cell wall of cyanobacterial cells lacks cellulose and their biomass is more rich in proteins, especially in filamentous diazotrophic species (Vargas et al., 1998). However, very little is still known about the identity of protein-utilizing microbial communities in soda lakes participating in cyanobacterial biomass mineralization. Recent studies identified two novel genera of aerobic extremely salt-tolerant alkaliphilic bacteria specialized on using various proteins as growth substrates, a gammaproteobacterium *Natronospira proteinivora* and *Natronotalea proteinilytica*, a representative of the phylum *Rhodothermaeota* (Sorokin et al., 2017a,b). Recently, a haloalkaliphilic anaerobic proteolytic clostridium, described as *Proteivorax tanatarense* gen. nov., sp. nov., was shown to utilize the cell proteins produced by a soda lake benthic cyanobacterium *Geitlerinema* sp. (Kevbrin et al., 2013). In addition, for hypersaline conditions, three natronoarchaeal species have been shown to produce haloalkali-stable proteases at salt-saturated conditions and high pH by members of the genera *Natronococcus*, *Natrialba*, and *Natronolimnobius* (Studdert et al., 2001; Selim et al., 2014; Derntl et al., 2015).

In this work, we describe phenotypic and genomic properties of a moderately salt-tolerant alkaliphilic aerobic protein-utilizing bacterium which developed in a stable co-culture with soda lake benthic filamentous cyanobacteria. The isolate belongs to a novel deep phylogenetic lineage within the recently suggested phylum *Balneolaeota*, a former part of the phylum *Bacteroidetes* (Hahnke et al., 2016; Munoz et al., 2016), forming a new genus and species candidate taxon “*Ca*. Cyclonatronum proteinivorum.”

## Materials and Methods

### Enrichment and Cultivation Conditions

An enrichment culture of filamentous benthic cyanobacteria was obtained from a biofilm developed in July 2011 on the surface of littoral sediments of the soda lake Bitter-3 (south Kulunda Steppe, Altai region, Russia; N51°40′/E79°53′). The brines total salinity was 90 g l^-1^ soluble carbonate alkalinity – 1.0 M and the pH 10.3. The enrichment culture was grown in 2 L Erlenmeyer flask containing 1 L medium mounted on a magnetic stirrer at 25°C and at ambient (day/night) light regiment. The basal mineral medium contained 1.3 M total Na^+^ at pH 10 with the following composition (g l^-1^): Na_2_CO_3_ – 57; NaHCO_3_ – 9; NaCl – 9, K_2_HPO_4_ – 1, KNO_3_ – 0.6, MgSO_4_ × H_2_O – 0.25, acidic trace metal solution (Pfennig and Lippert, 1966) – 1 ml. The fed-batch culture was maintained for 2 years aimed to produce biomass of the soda lake cyanobacteria. In several months the culture acquired a stable domination of two types of benthic filamentous haloalkaliphilic cyanobacteria with large and small cells identified as *Geitlerinema* sp. and *Nodosilinea* sp., respectively (Samylina et al., 2014).

Strain Omega was consistently developing as a heterotrophic satellite in several parallel cultures of haloalkaliphilic filamentous cyanobacteria enriched from a soda lake in *S-W* Siberia (Supplementary Figure S1). For isolation, a solid medium was prepared from the filter-sterilized cyanobacterial mineral medium, supplemented with sonicated and filter-sterilized cell-free extract of cyanobacteria after mixing 1:1 with 4% sterile agarose at 50°C. The inoculum was prepared from a stationary phase cyanobacterial culture after removing the cyanobacterial aggregates first by settling and the remaining suspended filaments – by a low-speed centrifugation. The inoculated plates were incubated up to 1 month in closed plastic bags at 25°C. A pure culture was isolated from a single colony after several rounds of restriking onto the solid medium. Further experiments with pure culture were performed in liquid salt media.

For standard cultivation and phenotypic characterization of strain Omega, a sodium carbonate-based medium buffered at pH 10 and containing 1 M total Na^+^ and casein peptone as substrate were used. For the salinity range (from 0.1 to 3 M total Na^+^, pH 10), the culture was pregrown at 1 M total Na^+^. An additional attempt to measure an absolute maximum of the salt tolerance was made afterward using a culture grown at maximum salinity in the first round. For the pH profiling, a range of pH from 6.5 to 11 with an increment of 0.5 unit was created using the following buffer systems containing 1 M total Na^+^: 0.08 M HEPES/0.05 M K-phosphate for pH from 6.5 to 8 and sodium bicarbonate-carbonate buffer system for pH 8-11. Growth (OD_600_) and the actual pH were monitored until the maximum OD values were reached. The temperature profile was measured at pH 10 and a total Na^+^ 1 M from 20 to 50°C with an increment of 5°C. Anaerobic growth either by fermentation or respiration with casein peptone carbon and energy source was tested in 10 ml cultures placed into 23 ml serum bottles closed with butyl rubber and made anoxic by 5 cycles of evacuation-flushing with sterile argon gas.

### Analytical Procedures

Biomass growth dynamics was followed by measuring optical density at 600 nm. Phase contrast microphotographs were produced with a Zeiss Axioplan Imaging 2 microscope (Göttingen, Germany). Pigments were extracted from wet cell biomass using 7:3 mixture of MeOH-aceton and 30 min vortexing. Absorption spectra were recorded on the UV-Visible diode-array HP 8453 spectrophotometer (Hewlett Packard, Amsterdam, Netherlands). The protease activity was tested qualitatively by diffusion-to-agar technique. For this, the culture supernatant was first passed through 0.22 μm syringe filter to remove residual cells and then 20 times concentrated using 20 ml Centricon tubes (Millipore) with 30 and 10 kDa membrane. The cell pellet was sonicated and the unbroken cells removed by 5 min centrifugation in 2 ml Eppendorf tube at 14,000 rpm, resulting in the cell-free extract fraction. 30 μl aliquats of each fraction were applied to wells cut into 1% casein agarose supplemented with sodium carbonate buffer containing 0.6 M total Na^+^ at pH 10. The plate was incubated for 72 h at 30°C and the hydrolysis zones were visualized by flooding with 10% (w/v) TCA solution.

### Activity of a Sodium-Translocating Proteorhodopsin in Washed Cell Suspensions

The cells were grown aerobically in medium containing 1 M total Na^+^ (0.8 M Na carbonates/0.2 M NaCl), pH 10 at 30°C and harvested by centrifugation. The cell pellet was washed three times in a salt solution containing 0.25 M Na_2_SO_4_ and 0.55 M K_2_SO_4_, and finally resuspended in the same solution at concentration of × 20 times the original culture. In the experiments with “minus sodium” the washing salt solution contained 0.8 M K_2_SO_4_. All the experiments were performed in a completely darkened thermostated room at 20°C. To measure the light-induced proteorhodopsin responses, 400 μl of the washed cell suspension was placed into a 500 μl-transparent plastic vial mounted on a magnetic stirrer and covered with a thermally insulating plate. This photocell was illuminated by a “cold” light using a custom-made illuminator OVS-1M equipped with two fiber-optic cables and halogen incandescent lamp KGM (9 V × 70 W) with the light flux in each fiber-optic cable of 17 W (Lytkarino Optical Glass Factory of optical glass and materials, Moscow region, Russia). The light-induced pH changes in the bacterial suspensions were continuously monitored with a glass InLab pH-microelectrode (Ø 3 mm, Mettler Toledo) connected to PHM250 ion analyzer (Radiometer Analytical SAS, France). The light-dependent alkalinization of the medium was taken as an indication of the Na^+^ extrusion (Inoue et al., 2013).

### Genome Sequencing and Annotation

The strain Omega genome was sequenced using both paired-end and mate-paired DNA libraries. Paired-end libraries were prepared using the Ultra-DNA library preparation kit (New England Biolabs, United States) according to manufacturer’s instructions to obtain mean library size of 500 bp. Mate-paired libraries were prepared with Nextera^TM^ Mate Pair Library Prep Kit (Illumina Inc., United States). Finally, one paired-end and three mate-paired libraries were sequenced with MiSeq^TM^ Personal Sequencing System (Illumina Inc., United States) resulting in 2 × 250 bp reads massive. After sequencing, all reads were subjected to stringent quality filtering with CLC Genomics Workbench 8.5 (Qiagen, Germany). After filtering, overlapping paired-end library reads were merged with SeqPrep tool^1^ resulting in 549,472 single merged reads and 178,732 read pairs with mean insert size of 602 bp. Mate paired reads were treated with NextClip tool (Leggett et al., 2014), resulting in 712,544 read pairs with mean insert size of 2233 bp. Reads were assembled with both ALLPATHS-LG (Butler et al., 2008) and SPADES 3.7.0 (Nurk et al., 2013) assemblers and refined manually using CLC Genomics Workbench 8.5 software (Qiagen, Germany), resulting in five high-quality contigs. Orientation of contigs and final filling of sequence gaps were made by PCR with outward-oriented primers to contigs termini and subsequent Sanger sequencing of resulting amplicons. Final average genome coverage of a single chromosome was 95.7 ×.

Gene prediction and primary annotation were performed with IMG/M ER System (Chen et al., 2017). Refining of the automated annotations and some particular predictions were done according to the protocol of Toshchakov et al. (2015). The annotated genome sequence of strain Omega has been deposited in the GenBank database under accession number CP027806. Corresponding Bioproject and Biosample IDs are PRJNA392178 and SAMN07284862, respectively.

### Phylogenetic Analysis

16S- and 23S-rRNA gene sequences of representatives of *Bacteroidetes* and its two recently proposed sister phyla *Rhodothermaeota* and *Balneolaeota*, as well as of *Chlorobi* and *Ignavibacteriae*, were downloaded from the Silva (SSU_126 & LSU_126) databases. Together with the 16S- and 23S-rRNA gene sequences of strain Omega, they were concatenated and aligned in MAFFT v. 7 (G-INS-i algorithm, Katoh and Standley, 2013). JModel Test was used to test the best evolutionary model, which appeared to be the General Time Reversible (GTR) model (*G + I*, 4 categories) (Nei and Kumar, 2000). The tree with the model and 1000 bootstrap resamplings was inferred in MEGA 6.0 (Tamura et al., 2013).

Sequences of 27 ribosomal proteins universally conserved in 146 bacterial species (Supplementary Table S1) representing almost all currently known phyla with completely or almost completely sequenced genomes were aligned using MAFFT v. 7 (E-INS-i algorithm, Katoh and Standley, 2013). Alignments were concatenated and the phylogenetic tree was inferred using MEGA 6.0 (Tamura et al., 2013) with LG evolutionary model and gamma-distributed site rates.

For phylogene analysis of rhodopsin proteins, sequences of all characterized rhodopsins were searched using BLAST with a CYPRO_0974 as a query and Uniprot and SwissProt databases (16.05.2018) as the references. Altogether, 45 sequences were aligned in MAFFT v. 7 (L-INS-i algorithm, Katoh and Standley, 2013). The tree was constructed in MEGA 6.0 (Tamura et al., 2013) with LG evolutionary model and gamma-distributed site rates.

Subtilases phylogeny was performed for all S8 endopeptidases family proteins, found in *in silico* translated Omega proteome as well as from the SwissProt database (30.03.2018). Only proteins with >250 aa from SwissProt were chosen including 327 of the total 341 S8 peptidases. After clustering using 70% sequence identity threshold in CD-HIT (Huang et al., 2010), 161 Swissprot sequences remained and together with Omega proteins 170 S8 peptidase members were aligned in MAFFT v. 7 (L-INS-i algorithm, Katoh and Standley, 2013). The tree was constructed in MEGA 6.0 (Tamura et al., 2013) with LG evolutionary model and gamma-distributed site rates.

The S51 family peptidases phylogeny was performed using two sequences of strain Omega (CYPRO_0608 and CYPRO_2145), five characterized cyanophycinases and peptidases E (both belong to S51 family) from SwissProt, twenty two sequences from Pfam (seed sequences of PF03575 (Peptidase_S51) domain) and 100 first best BLAST hits of CYPRO_0608 and CYPRO_2145 (50 each) from Uniprot. After deleting duplicates, 129 sequences were aligned in MAFFT v. 7 (L-INS-i algorithm, Katoh and Standley, 2013). The tree was constructed in MEGA 6.0 (Tamura et al., 2013) with LG evolutionary model and gamma-distributed site rates.

## Results and Discussion

### Isolation of Strain Omega

**FIGURE 1:**
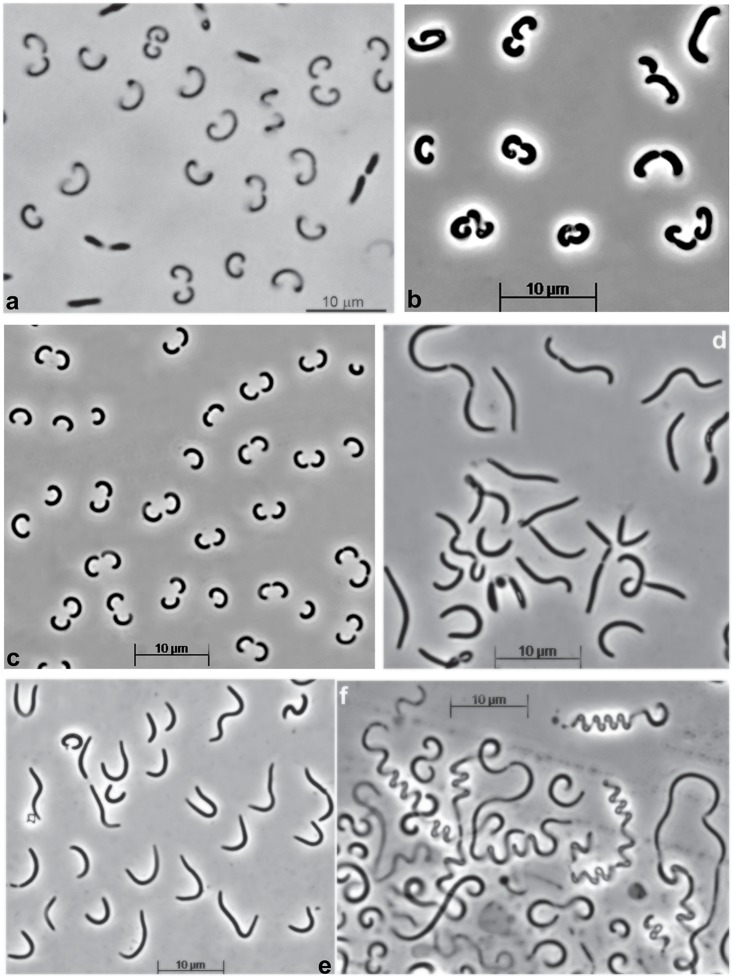
Cell morphology of strain Omega (phase contrast microphotographs) grow at pH 10 and 30°C in **(a)** a mix culture developing in supernatant of cyanobacterial enrichment from a soda lake, and **(b-f)** in pure culture. **(b)** Cells from colonies developing on solid medium with cyanobacterial cell extract as substrate; **(c,d)**, cells grown with albumin in liquid culture at 2 M Na^+^ and 1 M Na^+^, respectively; **(e,f)**, cells grown in liquid culture at 1 M Na^+^ with casein and caseinopepton, respectively.

Replicate enrichment cultures of filamentous benthic cyanobacteria from a soda lake in Kulunda Steppe (Altai, Russia) were developing in two phases. In the first phase, the cyanobacteria formed massive aggregated biomass layer floating on the top of incubation flask with no growth in the main medium body. In the second phase, the medium started to become turbid with pink coloration, while the cyanobacterial layer diminished and started to decompose (Supplementary Figure S1). We assumed that this pattern indicated a usual type of succession from a primary producer to heterotrophic degraders of the phototrophic biomass. Microscopic examination of the pink layer showed a domination of a bacterium with peculiar cell morphology in the form of semicircles with only a few other rod-shaped bacteria (Figure 1a). Since preliminary clone library (results are not shown) indicated that the dominant component belongs to an uncultured deep lineage bacteroidetes, attempts to isolate it in pure culture has been initiated using a solid medium containing sonicated cell extract from the cyanobacterial biomass as substrate. After a long incubation (3 weeks) small reddish colonies appeared which, after several rounds of resrtiking resulted in a pure culture, designated strain Omega, with a cell morphology resembling a dominant heterotrophic satellite (Figure 1b). However, the cell morphology varied depending on substrate and salinity, from semicircles to long “whip-like” cells (Figures 1c–f). The biomass was colored pink-orange due to a presence of pigments extractable with a mixture of methanol-aceton (Supplementary Figure S2), most probably belonging to carotenoids.

### Basic Physiology of Strain Omega

**FIGURE 2:**
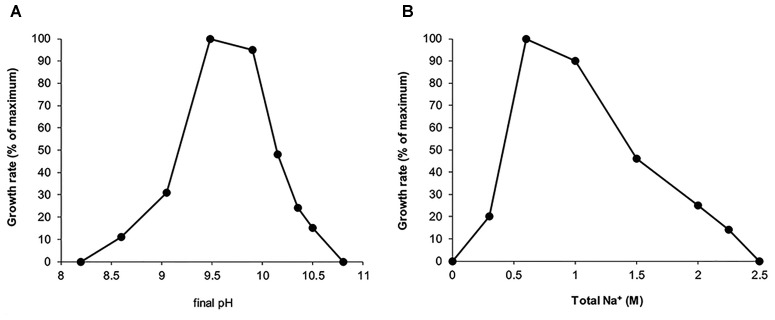
Influence of pH at 1 M total Na^+^ **(A)** and influence of total Na^+^ in the form of carbonates at pH 10 **(B)** on growth of strain Omega with caseinopepton. The actual pH values are presented on the X axis. The results are mean from a duplicate with the maximum deviation of less than 20%.

Further work with the isolate demonstrated that it belongs to a functional group of obligate aerobic protein degraders. Best growth was observed with peptone from casein, but also other peptones, such as meat and soy, and yeast extract can serve as growth substrates. Among the proteins, the isolate was able to grow with heat-sterilized casein, bovine serum albumin and gelatin. The cells grown with casein showed a presence of cell-bound protease active at salt concentration from 0.2 to 1 M Na^+^ and with a pH optimum of 9–10 (Supplementary Figure S3). Sugars (glucose, fructose, galactose, mannose, lactose, xylose, sucrose, trehalose, cellobiose, maltose) and sugar polymers (such as starch, amorphous cellulose, chitin, pectin, alginate) did not support growth. Fermentative growth with peptone from casein and glucose was also not observed, as well as with added electron acceptors, such as nitrate, nitrite, sulfur, thiosulfate and fumarate. Strain Omega is a moderately salt-tolerant obligate alkaliphile with a pH range for growth from 8.5 to 10.5 (optimum at 9.5–10) and salt range at pH 10 from 0.3 to 2 M Na^+^ (Figure 2). In contrast to most of the moderately salt-tolerant bacterial isolates from soda lakes, strain Omega required cloride ion both for optimal growth and storage of the grown culture.

### General Genome Properties and Genome-Based Phylogeny

**FIGURE 3:**
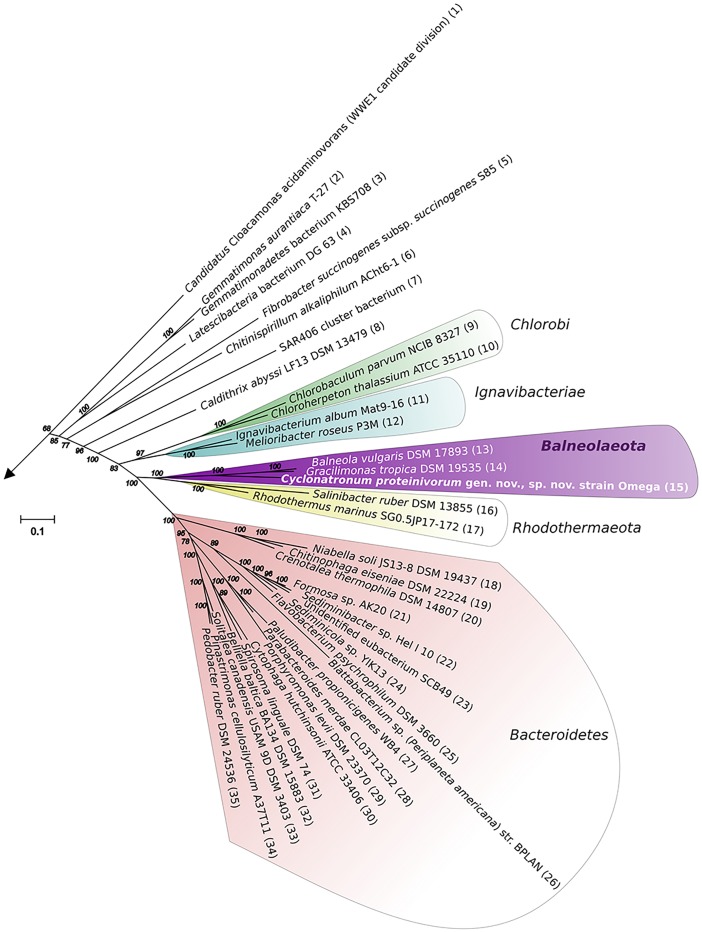
27 concatenated conservative proteins-based phylogenetic analysis. The tree was constructed using Maximum-Likelihood method. The tree with the highest log likelihood is shown. The bootstrap values (100 replicates) are shown next to the branches. The tree is drawn to scale, with branch lengths measured in the number of substitutions per site. The analysis involved 136 amino acid sequences and 4318 positions. All positions with less than 95% site coverage were eliminated. The unrooted tree was generated in MEGA6 (Tamura et al., 2013). Only FCB superphylum representatives are shown. The sequences were obtained from IMG and have the following accession numbers: (1), 642560256; (2), 643773439; (3), 2578408359; (4), 2656359422; (5), 646369571; (6), 2638713609; (7), 265102961; (8), 2562178319; (9), 642718814; (10), 642716968; (11), 2514222721; (12), 2517204605; (13), 2515844915; (14), 2515866577; (15), 2695201269; (16), 637844027; (17), 2505982187; (18), 2506845178; (19), 2635114424; (20), 2601542687; (21), 2532033211; (22), 2561440247; (23), 641144478; (24), 2623579954; (25), 2623246297; (26), 646377621; (27), 649775995; (28), 2531043488; (29), 2515619891; (30), 638073153; (31), 646498001; (32), 2509560651; (33), 2509579588; (34), 2671580424; (35), 2677155390.

**FIGURE 4:**
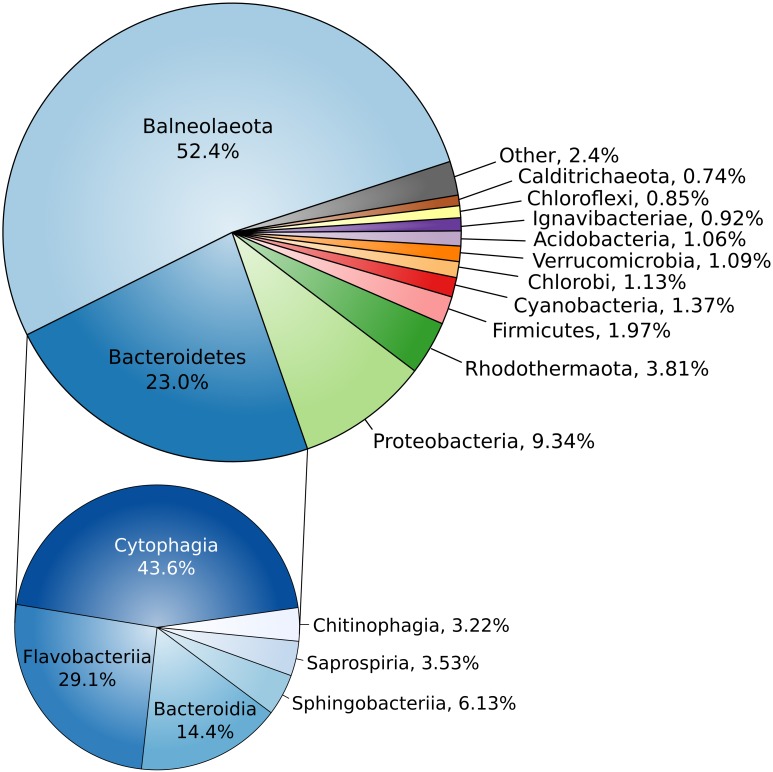
Blast-hit distribution of proteins. Taxonomic distribution of *in silico* translated proteome of strain Omega best BLAST hits. All hits are classified by phyla (top) and *Bacteroidetes* hits are classified by classes (bottom).

Assembled complete genome of strain Omega consists of 4294247 bp and does not contain any extrachromosomal elements (Supplementary Figure S4). Genome harbors forty three tRNA genes and three ribosomal operons with identical structure: 16S – tRNA-Ile – tRNA-Ala – 23S – 5S. Average G + C content is 51.39%. Genome contains 3284 protein-coding genes, 2349 (70.35%) of which were assigned with function. Two concatenated trees, based on 16S + 23S rRNA genes (Supplementary Figure S5) and on 27 conservative ribosomal proteins (Figure 3) placed strain Omega within the recently proposed phylum *Balneolaeota* (Hahnke et al., 2016) as a separate deep lineage. Distribution of Best BLAST Hits of *in silico* translated Omega proteome revealed strong domination of the proteins mostly related to the members of *Balneolaeota* (Figure 4; Supplementary Figure S6). Surprisingly, the number of hits, affiliated with the nearest phylum *Rhodothermaeota* was relatively low in comparison with *Bacteroidetes sensu stricto* and *Proteobacteria*. Yet, this is most probably due to a quantitative bias from the number of analyzed genomes of each phyla. 16S rRNA genes identity, calculated during pairwise alignment (BLAST) with validly published representatives of *Balneolaeota* was less than 88% with *Balneola vulgaris* strain 13IX (Urios et al., 2006) as the closest relative (87.7%), indicating that the soda lake isolate is forming a deep phylogenetic lineage at the level of a novel family. Furthermore, culture-independent surveys demonstrated a presence of two groups of clones related to strain Omega found previously in a low salt alkaline Lonar lake in India (clone SA_226, sequence identity 97%) (Antony et al., 2014) and a hypersaline soda lake Mono in California (clone ML602J-34, sequence identity 92%) (Humayoun et al., 2003).

### Genome-Based Metabolic Reconstruction

Results of the analysis of COG distribution performed by IMG-ER (Chen et al., 2017) supported phenotypic observations on the proteolytic lifestyle of strain Omega. High number of genes encoding proteins, participating in amino acid transport and metabolism (172 proteins, 8.56% of total proteome), as opposed to a lesser proportion of proteins, involved in carbohydrate transport and metabolism – 4.96%, has been observed in the genome which is significantly less than in *Rhodothermus marinus*, known for its active carbohydrate utilization (Supplementary Table S2). A comparatively high number of proteins involved in defense mechanisms (4.11%, the highest percentage among complete genomes of *Balneolaeota* and *Rhodothermaeota*) seems reasonable for an organism closely associated with cyanobacteria. High abundance and expression levels of proteins participating in defense mechanisms has recently been reported for cyanolytic bacterial heterotrophs (Osman et al., 2017).

It should be also noted, that among analyzed representatives of *Balneolaeota* and *Rhodothermaeota*, the genome of strain Omega encodes the highest number of proteins with only general function prediction (10.47%) and not assigned to any cluster of orthologous groups (44.95%), which reflects its deep-lineage phylogenetic position. The COG-based hierarchical clustering shows the Omega proteome is only distantly related to the genome-sequenced representatives of *Balneolaeota* and *Rhodothermaeota*. In contrast, hierarchical clustering by functional categories revealed that strain Omega located close to a *Balneolaeota* representative *Gracilimonas tropica*, which has also been isolated from cyanobacterial biomass (Supplementary Figure S6). This is an indication that despite their distinct phylogenetic positions both microorganisms independently adapted to their cyanobacteria-associated lifestyle. A recent metagenomic reconstructions from the Kulunda Steppe hypersaline soda lake brines also showed a prominent presence of a *Balneola*/*Gracilimonas*-related lineage there (T5-Br10_g13) (Vavourakis et al., 2016).

### Genome Plasticity and Genomic Islands

In prokaryotes, lateral gene transfer is the most widespread and universal mechanism of rapid adaptation to environmental changes (Madsen et al., 2012). Such adaptations might be particularly important for highly dynamic environments of hypersaline soda lakes in Central Asia, subjected to seasonal algal blooms, periods of extreme evaporation and dilution and a large annual temperature fluctuations. Omega genome was inspected for LGT events with Seqword Island Sniffer program, searching for atypical tetranucleotide usage (Bezuidt et al., 2009) and Island Viewer 3 package (Dhillon et al., 2015), implementing IslandPath-DIMOB (Waack et al., 2006), and SIGI-HMM, algorithms (Hsiao et al., 2003). 22 putative genomic islands (GI) of 377 kb total length were predicted (Supplementary Figure S5 and Supplementary Table S3). Most of genes of predicted GIs encoded proteins with unknown functions (55%), mobile element proteins (16%), proteins of defense systems (14%), which is typical for horizontally transferred genes. However, closer inspection of The Best BLAST hits of the majority of genes encoding these proteins showed that most of them have the best blast hits in closely related classes: *Balneolia* (24%), *Flavobacteria* (13%), or *Cytophagia* (11%), representatives of which could also be found in similar environments. Omega genome harbors 52 presumably functional or inactivated transposase-containing mobile elements, occupying totally over 67 Kbp. Analysis of transposase diversity with *ISSaga* showed that they belong to 12 different IS families. Two families, IS1380 and IS66 were not present either in *Rhodothermaeota* or *Balneolaeota* genomes, making them unique for the novel lineage within these Bacteroidetes-related phyla. While IS1380 elements contained only partial transposases, all four IS66 were complete, consisting of three ORFs which produce three functional proteins by translation coupling mechanism (Han et al., 2001). Interestingly, IS66 elements are mostly widespread in *Proteobacteria*, while only several IS66 lineages were detected in *Bacteroidetes/Clorobi* hosts (Gourbeyre et al., 2010).

### Elements of Sodium-Dependent Bioenergetics

**FIGURE 5:**
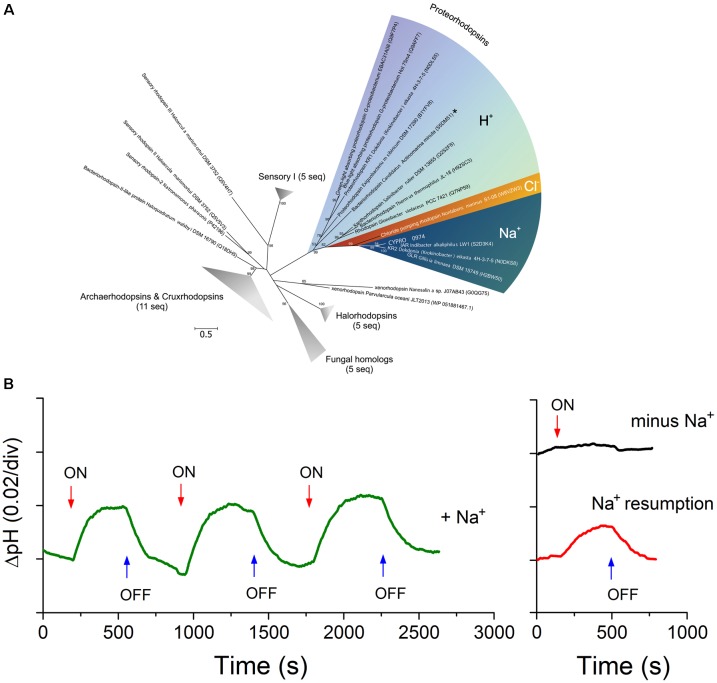
A presence of Na^+^-translocating light-driven primary ion pump in strain Omega. (**A**) Phylogenetic analysis of a rhodopsin protein encoded by the Omega genome. The tree was constructed using Maximum-Likelihood method. The tree with the highest log likelihood is shown. The bootstrap values (1000 replicates) are shown next to the branches. The tree is drawn to scale, with branch lengths measured in the number of substitutions per site. The analysis involved 45 amino acid sequences and 206 positions. All positions with less than 95% site coverage were eliminated. The unrooted tree was generated in MEGA6 (Tamura et al., 2013). CYPRO_0974 is in bold. (**B**) Prove of functionality of the light-dependent primary sodium pump using washed cells of strain Omega. Light-induced alkalinization was registered by a pH microelectrode. The measuring medium (left graph) contained 0.25 M Na_2_SO_4_ and 0.55 M K_2_SO_4_. The “sodium free” medium (right graph) contained 0.8 M K_2_SO_4_. The illumination switches are indicated by arrows.

The proteorhodopsin gene *CYPRO_0974* identified in Omega genome has the *NDQ* motif unique for the Na^+^-translocating proteorhodopsin (NaR) recently discovered in a few members of salt-tolerant *Bacteroidetes* (Inoue et al., 2013; Kato et al., 2015; Pinhassi et al., 2016). It formed an independent lineage within the cluster of NaR with confirmed function (Figure 5A). Washed cells of strain Omega exhibited a light-induced alkalinization of the Na^+^-containing medium (Figure 5B), similar to the marine *Krokinobacter eikastus* (Inoue et al., 2013). The effects could be explained by functioning of a NaR or a halorhodopsin (HR). However, HR does not pump SO_4_^2-^ and since in this experiment we only used sodium/potassium sulfate in the incubation buffer, the HR possibility is eliminated. Moreover, when the sodium sulfate was replaced by potassium sulfate, the light-induced alkalinization in the Omega cell suspension ceased (Figure 5B). This differentiated the Omega proteorhodopsin from the xenorhodopsin behavior (Inoue et al., 2016; Shevchenko et al., 2017). Introduction of sodium ions in the cell suspension reactivated the Omega Na^+^-proteorhodopsin. Thus, the *in vivo* experiments demonstrated the expression of an active light-dependent primary sodium pump in strain Omega, which is only a second (to a low salt-tolerant *Indibacter alkaliphilus*) example of a haloalkaliphile from soda lakes with confirmed functional NaR. Taking into account that the pumping activity of NaR in the native cell suspensions of Omega is close to that of *K. eikastus* (Inoue et al., 2013), the Omega NaR can be rather assigned to a true primary ion pump than to a sensor protein. Thus, we can assume that it has an important role as an energy transducer. Such a pump might be profitable in a bacterial species associated with benthic cyanobacteria living at oxygen-limiting conditions on the surface of sulfidic sediments. At these conditions (O_2_ limitation + high alkalinity + unlimited sodium), Omega-like heterotrophic satellites should be at an advantage utilizing an O_2_-independent way of solar energy transduction by NaR, as it would allow such organisms to build up an additional sodium-based transmembrane difference of electrochemical potential (Banciu and Muntyan, 2015).

Another putative mechanism of Na^+^ export is based on the action of a multi-subunit Na^+^/H^+^ antiporter Mrp (Swartz et al., 2005), encoded in the Omega genome by Cypro_0615-0621. No genes for Rnf Na^+^/H^+^ exporter complex were found.

### Adaptation to Salinity

The genome of strain Omega encodes a classical pathway for synthesis of the typical organic osmolyte glycine betaine including oxidation of choline to betaine aldehyde and its further oxidation to betaine (Roberts, 2005). First step might be performed by either ferredoxin-dependent choline monooxygenase CYPRO_1993 or choline dehydrogenase CYPRO_1995. Second step, might be performed by one of five aldehyde dehydrogenases predicted in the Omega genome (CYPRO_1155, CYPRO_1282, CYPRO_1289, CYPRO_2064 and CYPRO_2065) among which CYPRO_2065 is being the most reliable candidate according to search of HMM profile of curated SwissProt betaine aldehyde dehydrogenase sequences as a query. The genes for sucrose and trehalose biosynthesis, that are often found in halophilic prokaryotes (as secondary) and eukaryotes (as primary, together with sugar alcohols) osmoprotectants (Oren, 2013; Gostinčar et al., 2011) have also been identified in the Omega genome. On the other hand, the genes, encoding proteins, related to ectoine biosynthesis, another osmolyte commonly found in halophilic and halotolerant bacteria, were not identified in the Omega genome.

### Central Metabolism

The genome analysis revealed a presence of all genes encoding glycolysis/gluconeogenesis and TCA cycle enzymes. While no growth on sugars was detected, glycolysis/gluconeogenesis most probably takes a course on the direction of synthesis of glucose-1-phosphate, which is being a substrate for intracellular oligo- and polysaccharides biosynthesis. All genes, encoding proteins for glycogen biosynthesis were found. Surprisingly, relatively high number of CAZymes-coding genes (Lombard et al., 2014) were also found. Among them the most numerous families were glycosidases GH13, GH74, GH23, GH3, GH17, carbohydrate esterases CE1 and CE10 and polysaccharide lyases PL22. While this is quite common in marine Bacteroidetes associated with algae (Kappelmann et al., 2018), in the proteolytic strain Omega a potential for polysaccharide hydrolysis seems to be unnecessary. It is possible, however, that some of these (e.g., amylase-related proteins of the family GH13) are involved in intracellular oligo- and polysaccharide turnover as well as extracellular polysaccharide synthesis. Finally, one cannot exclude the probability of hydrolysis of unknown oligo- and polysaccharides (possibly of complex nature, e.g., components of cyanobacterial cell walls or extracellular matrix), that have not been tested during growth experiments.

According to the IMG ER pipelines, predicting amino acids auxotrophy/eutrophy, strain Omega is an auxotroph to the number of amino acids (arginine, histidine, isoleucine, leucine, lysine, phenylalanine, proline, serine, tyrosine, and valine) and apparently dependent on the external source, in accordance with its key physiology. Its genome contains 134 genes encoding proteins (Supplementary Figure S5 and Supplementary Table S4) of known MEROPS (Rawlings et al., 2014) families. Most of them are peptidases, yet the minor part represents peptidase homologs, lacking peptidase activities. Among the MEROPS enzymes, 51 were predicted to be extracellular using SinalP and SecretomeP prediction servers. Of these, the most represented families were S9 (18 total, 11 secreted proteins), S8 (all 9 are secreted proteins), S12 (5 total, 3 secreted proteins) and M28 (6 total, 4 secreted proteins). While currently known S8 and M28 peptidases are mainly represented by exo- (amino- or carboxy-) peptidases, the majority of S9 family of peptidases (subtilases) act as endopeptidases, being involved in hydrolysis of extracellular peptides and proteins. Phylogenetic analysis (Supplementary Figure S7) showed that Omega subtilases formed three distinct clusters, all of which are distant from biochemically characterized relatives. All four Omega S12 family proteins (CYPRO_0566, CYPRO_2116, CYPRO_2188, CYPRO_3057) have beta-lactamases and related proteins among the nearest characterized homologs, such as, for example, MlrB microcystine defensive system of *Sphingomonas* sp. (Bourne et al., 2001), suggesting their involvement in defense mechanisms, apparently beneficial for a satellite of cyanobacteria, that are known to synthesize various toxins. Yet, the mechanisms of this defense are vague, for example, the only currently known mechanism of aerobic degradation of cyanobacterial microcystine (Li et al., 2017) relies on four mlrA-D proteins, while Omega genome encodes only distant homologs of MlrB and no detectable homologs of other Mlr proteins. Furthermore, two peptidases of S51 family were found (CYPRO_0608 and CYPRO_2145) to be encoded in the Omega genome. The family includes cyanophycinases – exopeptidases, involved in cyanophycin (multi-L-arginyl-poly-L-aspartic acid, a cyanobacterial storage compound) degradation. Phylogenetic analysis (Supplementary Figure S8) showed that the Omega S51 peptidases fell into two distinct clusters. Both clusters have cyanobacterial representatives close to their nodes indicating independent HGT from different cyanobacteria to opportunistic bacteria, co-habiting with them or vice versa. Within the clusters, both Omega cyanophycinases have the nearest homologs from representatives of the FCB superphylum (Kublanov et al., 2017). Similarly to their close homologs from *Salinivirga cyanobacteriivorans* – a cyanobacteria-feeding *Bacteroidetes* member (Ben Hania et al., 2017), two Omega proteins were structurally different: CYPRO_2145 is being a monomeric protein, while CYPRO_0608 has an additional Por secretion system domain. However, the genomic context of *CYPRO_0608* and *CYPRO_2145* differs from their *S. cyanobacteriivorans* counterparts implying another mechanism of action.

A BLASTp of the bacteriocin sequences from BACTIBASE (Hammami et al., 2010) against the *in silico* translated Omega proteome resulted in 20 proteins, homologous to halocin, carocin, zoocin, colicin, linocin, helveticin, aerocin, dysgalacticin and pyocin. Manual verification revealed that at least half of them indeed are being bacteriocins and the most numerous (5 coding sequences) were zoocins – M23 peptidases, responsible for peptidoglycan cross-bridges degradation (Gargis et al., 2009). However, a search for secondary metabolite biosynthesis genes in the Omega genome using AntiSMASH software (Weber et al., 2015) did not reveal clusters for bacteriocin biosynthesis implicating putative novel biosynthetic pathways.

## Conclusion

Overall, strain Omega represents a first example of an aerobic haloalkaliphilic protein-utilizing bacterium associated with a filamentous cyanobacterial bloom in hypersaline soda lakes. Its most prominent features include a peculiar cell morphology; the ability to utilize various proteins as carbon and energy source; a possession of an active light-dependent sodium-translocation primary ion pump representing a new lineage in the proteorodopsin family and deep-lineage phylogeny on the level of a new species-genus-family within the recently proposed phylum *Balneolaeota*. The genome analysis mostly confirmed the observed phenotypic properties, including proteolytic aerobic heterotrophic and haloalkaliphilic lifestyle. On the other hand, some of the functional genomic content (a presence of genes encoding polysaccharide-degrading GH family enzymes) was not validated by the *in vivo* tests. Furthermore, based on the overall results of phenotypic and phylogenomic analysis, strain Omega is suggested to form a novel candidate genus and species “*Candidatus* Cyclonatronum proteinivorum” in the phylum *Balneolaeota*.

## Author Contributions

DS isolated the culture and made its phenotypic characterization. MM tested cells for the activity of sodium rhodopsin. ST and AK sequenced and annotated the genome. IK, ST, and MM made phylogenetic analysis. All authors participated in writing the manuscript.

## Conflict of Interest Statement

The authors declare that the research was conducted in the absence of any commercial or financial relationships that could be construed as a potential conflict of interest.

## Footnotes

1 https://github.com/jstjohn/SeqPrep
