# Supplementary material for: Phenotypic and Genomic Properties of a Novel Deep-Lineage Haloalkaliphilic Member of the Phylum Balneolaeota From Soda Lakes Possessing Na+-Translocating Proteorhodopsin
Source: Front Microbiol. 2018 Nov 13;9:2672. doi: 10.3389/fmicb.2018.02672 (PMC6243061; doi:10.3389/fmicb.2018.02672)
Supplement: Supplementary file 2 [file Data_Sheet_1.pdf]

Supplementary data to:

Phenotypic and genomic properties of "Cyclonatronum proteinivorum", a haloalkaliphilic member of the phylum *Balneolaeota* from soda lakes possessing Na^+-translocating proteorhodopsin

Dimitry Y. Sorokin[1,2*], Maria S. Muntyan[3], Stepan V. Toshchakov[1], Aleksei Korzhenkov[4], Ilya V. Kublanov[1]

[1] *Winogradsky Institute of Microbiology, Research Center of Biotechnology of the Russian Academy of Sciences, Moscow, Russia*

[2]*Department of Biotechnology, Delft University of Technology, Delft, The Netherlands*

[3]*Lomonosov Moscow State University, Belozersky Institute of Physico-Chemical Biology, Moscow, Russia*

[4]*Immanuel Kant Baltic Federal University, Kaliningrad, Russia*

Supplementary Tables (tables S1, S3 and S4 are presented in a separate Excell file)

Table S1. 27 conservative proteins (in COG numbering) in 146 genomes from IMG used for phylogenetic analysis.

Table S2. Distribution of COG function categories in *in silico* translated proteomes of strain Omega and related microorganisms.

Table S3. Genomic islands identified in the genome of strain Omega.

Table S4. Repertoire of peptidases genes identified in the genome of strain Omega.

Supplementary Figures

Fig. S1. Phototrophic enrichment culture from Kulunda Steppe soda lake Bitter-3 containing benthic haloalkaliphilic cyanobacteria (*Geitlerinema* sp. and *Nodosilinea* sp. in the top layer and a dense Omega-dominated heterotrophic community feeding on the organic carbon produced by the cyanobacterial biomass.

Fig. S2. Absorption spectrum of a red pigment(s) extracted with MeOH-acetone (3:7 v/v) from cells of strain Omega grown in the dark with casein pepton at pH 10 and 1 M total Na^+.

Fig. S3. Proteolytic activity in cell fractions of strain Omega, grown on casein at pH 10 and 1 M total Na^+. The activity was visualized by agar diffusion method with casein as substrate, sample volume 30 μl, incubation time 48h at 30°C. The top panel shows influence of pH at 0.6 M total Na^+, the bottom panel – influence of soda at pH 10. The top well contains cell-free extract (containing soluble cytoplasmic and insoluble cell wall and cell membrane proteins with a total protein load of 120 μg); the two bottom wells – concentrated culture supernatant containing concentrated secreted protein fraction > 30 kD (left spot) and >10 kDa (right spot) with total load of 15 and 12 μg, respectively.

Fig. S4. Chromosome map of strain Omega. From inside to outside:

- GC-skew calculated with 10000bp sliding window with 1000 bp step. Negative values are shown in light orange, positive values are shown in light blue;

- GC-content bias calculated with 10000bp sliding window with 1000 bp step. Values smaller than 50% are shown in orange, values bigger than 50% are shown in purple;
- rRNA operons (green) and genomic islands (pale yellow);
- negative strand CDSs. Predicted peptidase-coding genes highlighted with blue, transporter-coding genes are highlighted with red. tRNA are highlighted with dark blue.
- positive strand CDSs. Peptidase and transporter genes are highlighted as for negative strand.
- genomic coordinates.

Fig. S5. 16S-23S rRNA concatenated genes phylogenetic analysis of strain Omega. The tree was constructed using Maximum-Likelihood method. The tree with the highest log likelihood is shown. The bootstrap values (1000 replicates) are shown next to the branches. The tree is drawn to scale, with branch lengths measured in the number of substitutions per site. The analysis involved 62 nucleotide sequences and 4039 positions. All positions with less than 95% site coverage were eliminated. The tree was generated in MEGA6 (Tamura et al., 2013). The strain Omega sequences are in bold. *Caldithrix abyssi* strain LF13 was used as an outgroup.

Fig. S6. COG FC vs COG-based hierarchical clustering of complete publicly available *in silico* proteomes of *Balneolaeota* and *Rhodothermaeota: Balneola vulgaris* DSM 17893, *Gracilimonas tropica* DSM 19535, *Rhodothermus marinus* R-10, *Salinibacter ruber* M8, *Salisaeta longa* DSM 21114 and *Cyclonatronum proteinivorum* Omega. Genome clustering was performed using corresponding tool in IMG-ER JGI MER (Chen et al., 2017).

Fig. S7. Subtilases (S8 peptidases) phylogenetic analysis. The tree was constructed using Maximum-Likelihood method. The tree with the highest log likelihood is shown. The bootstrap values (100 replicates) are shown next to the branches. The tree is drawn to scale, with branch lengths measured in the number of substitutions per site. The analysis involved 170 amino acid sequences and 221 positions. All positions with less than 95% site coverage were eliminated. The unrooted tree was generated in MEGA6 (Tamura et al., 2013). Nine subtilases from strain Omega are in bold.

Fig. S8. Phylogenetic analysis of the family S51 peptidases. The tree was constructed using Maximum-Likelihood method. The tree with the highest log likelihood is shown. The bootstrap values (100 replicates) are shown next to the branches. The tree is drawn to scale, with branch lengths measured in the number of substitutions per site. The analysis involved 129 amino acid sequences and 171 positions. All positions with less than 95% site coverage were eliminated. The unrooted tree was generated in MEGA6 (Tamura et al., 2013). Two subtilases from strain Omega are in bold.

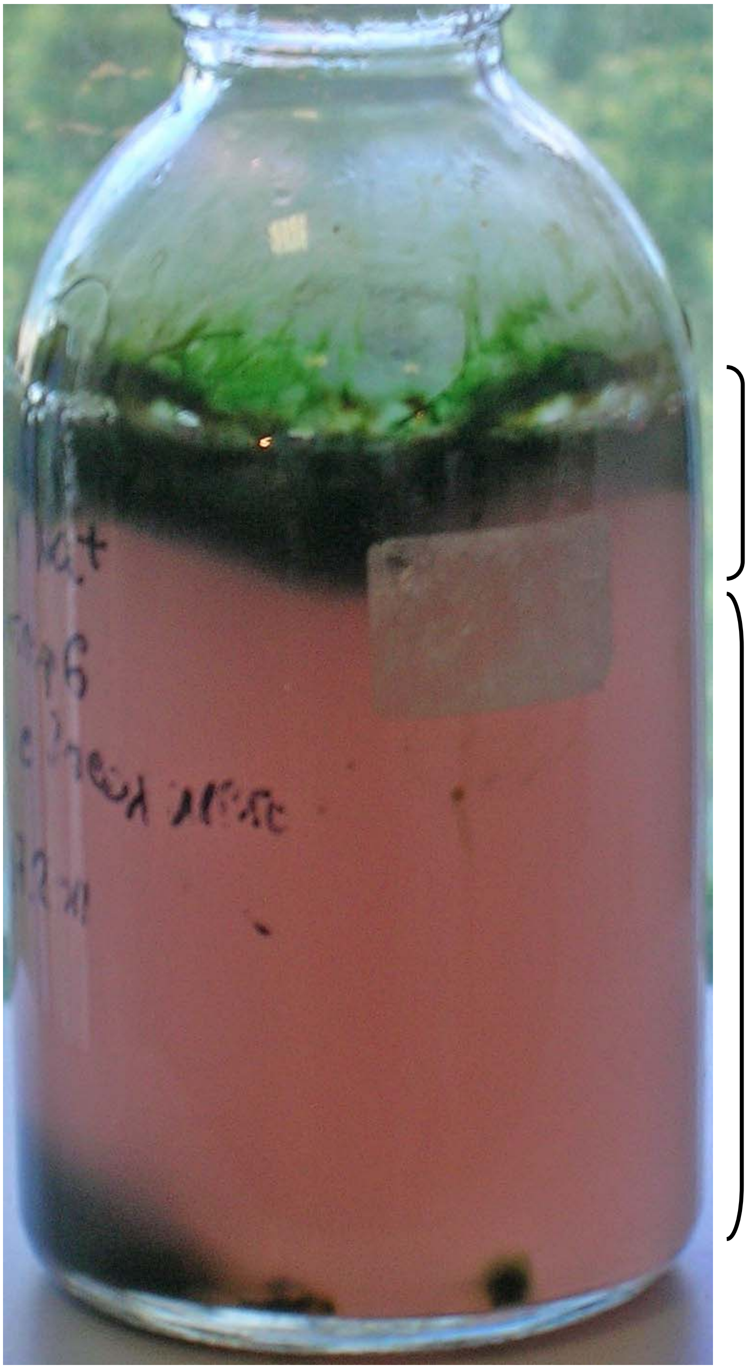

Supplementary fig.S1 [illegible]

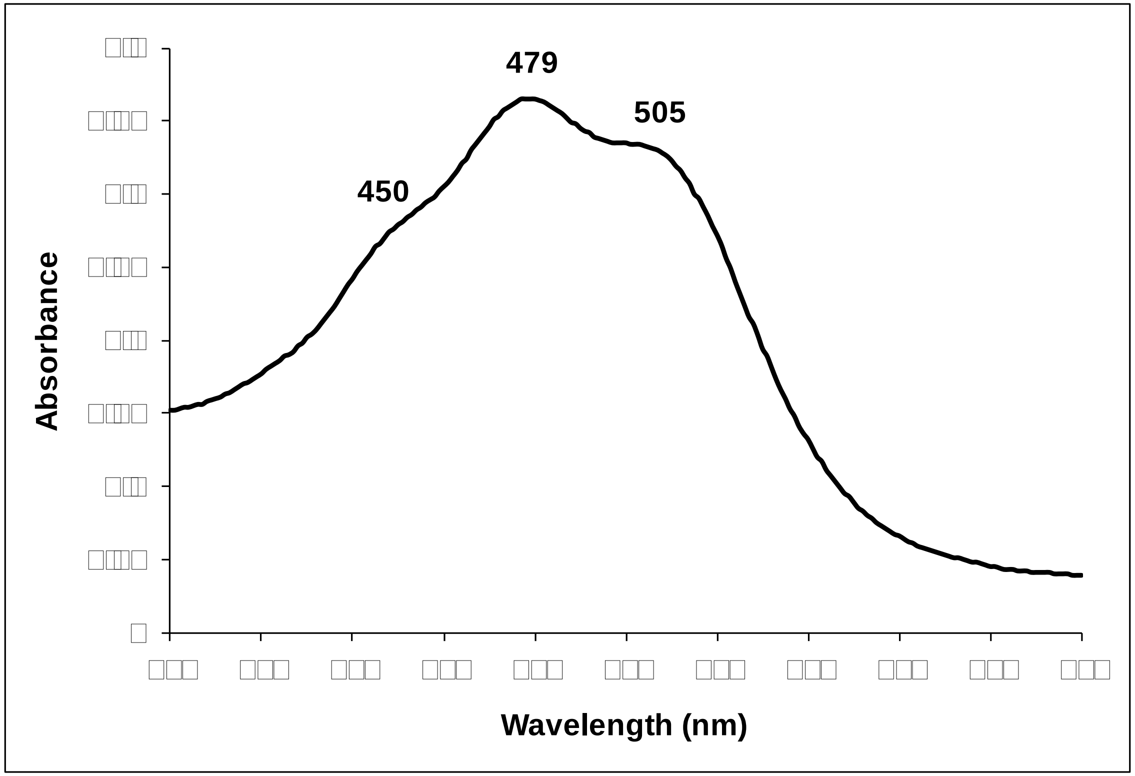

Supplementary Fig. S2. A [illegible]

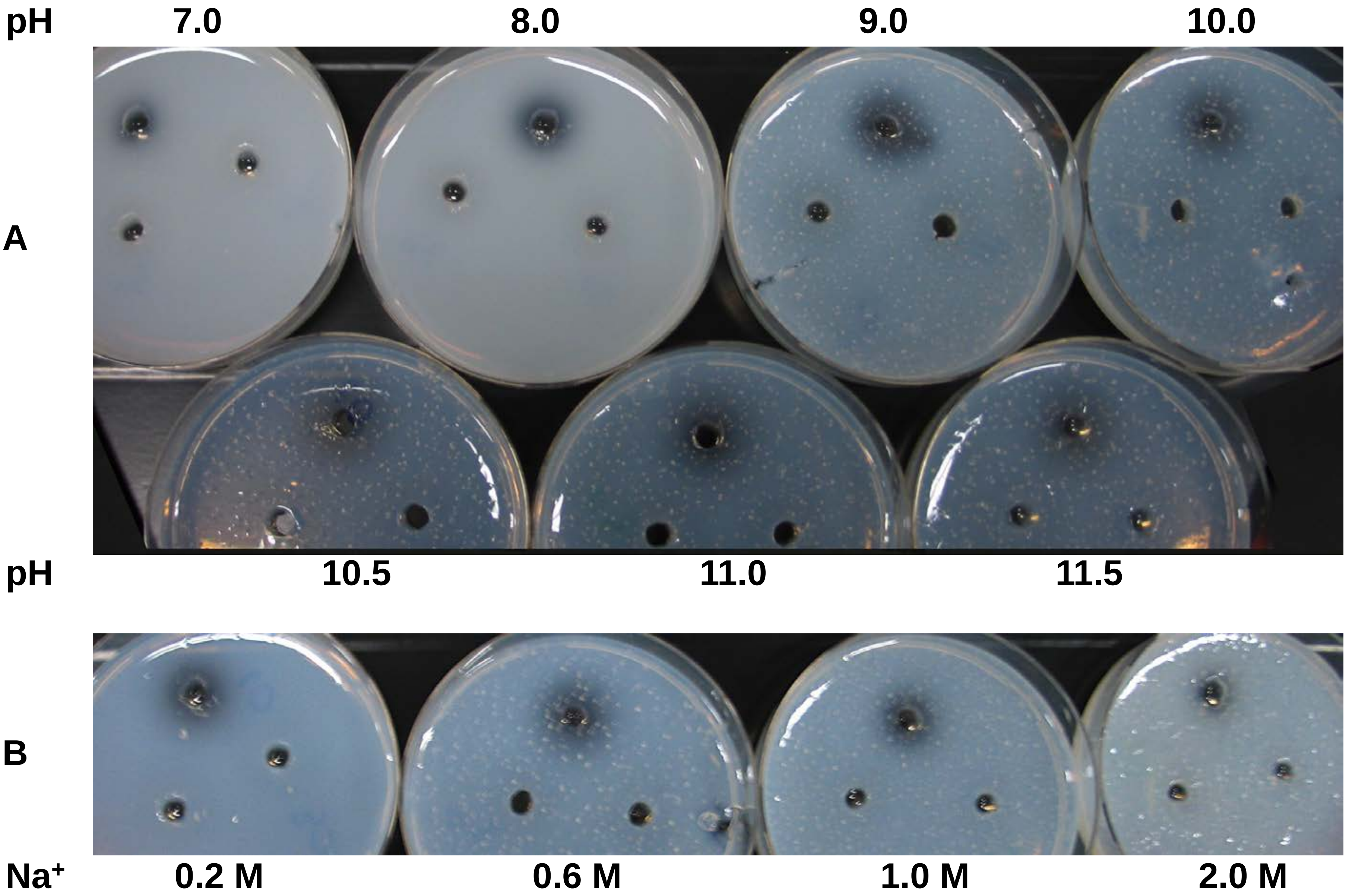

Supplementary Fig. S3 [illegible]

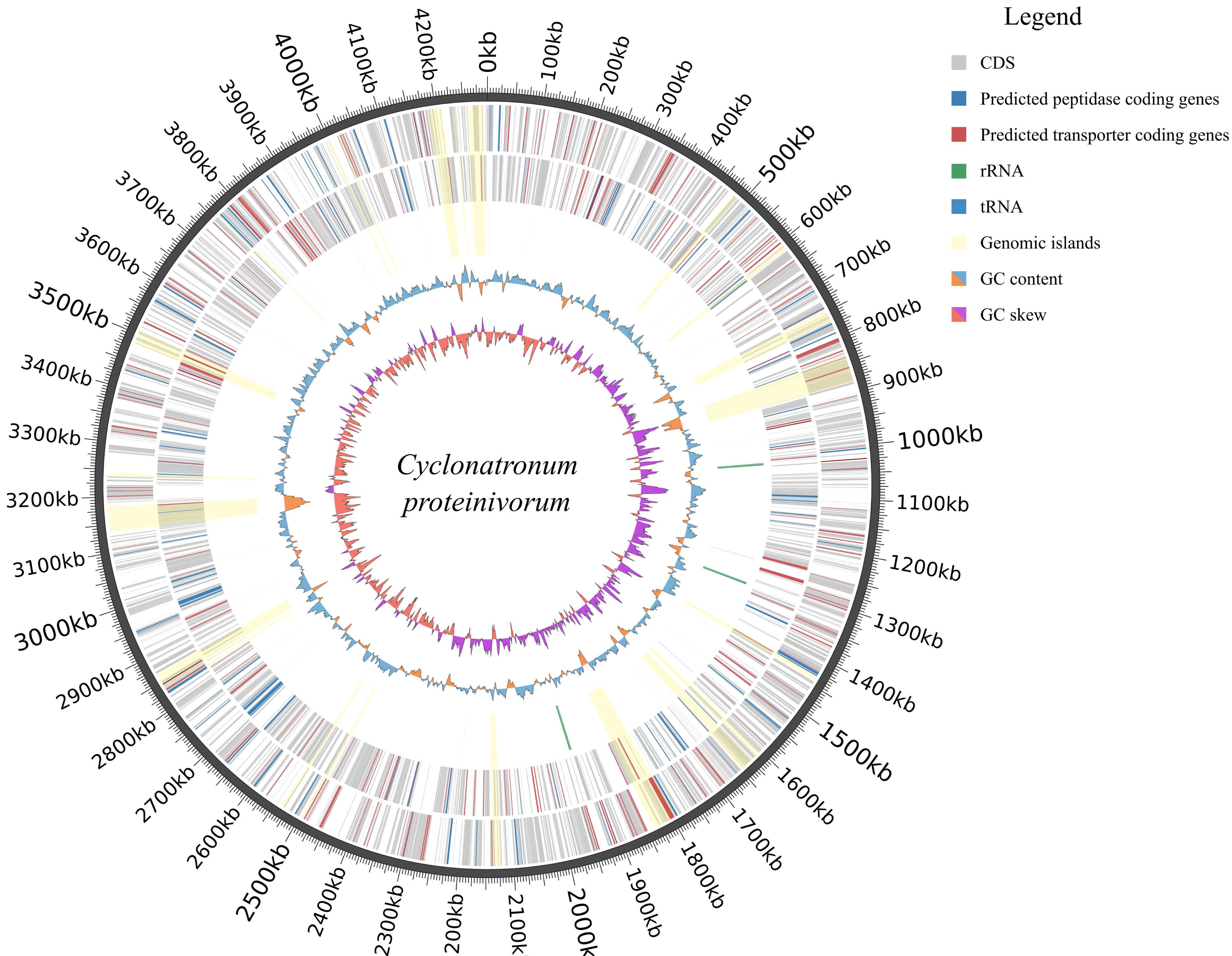
Legend
CDS
Predicted peptidase coding genes
Predicted transporter coding genes
rRNA
tRNA
Genomic islands
GC content
GC skew
Cyclonatronum proteinivorum
0kb
100kb
200kb
300kb
400kb
500kb
600kb
700kb
800kb
900kb
1000kb
1100kb
1200kb
1300kb
1400kb
1500kb
1600kb
1700kb
1800kb
1900kb
2000kb
2100kb
2200kb
2300kb
2400kb
2500kb
2600kb
2700kb
2800kb
2900kb
3000kb
3100kb
3200kb
3300kb
3400kb
3500kb
3600kb
3700kb
3800kb
3900kb
4000kb
4100kb
4200kb

Fig.S4

Fig. S5

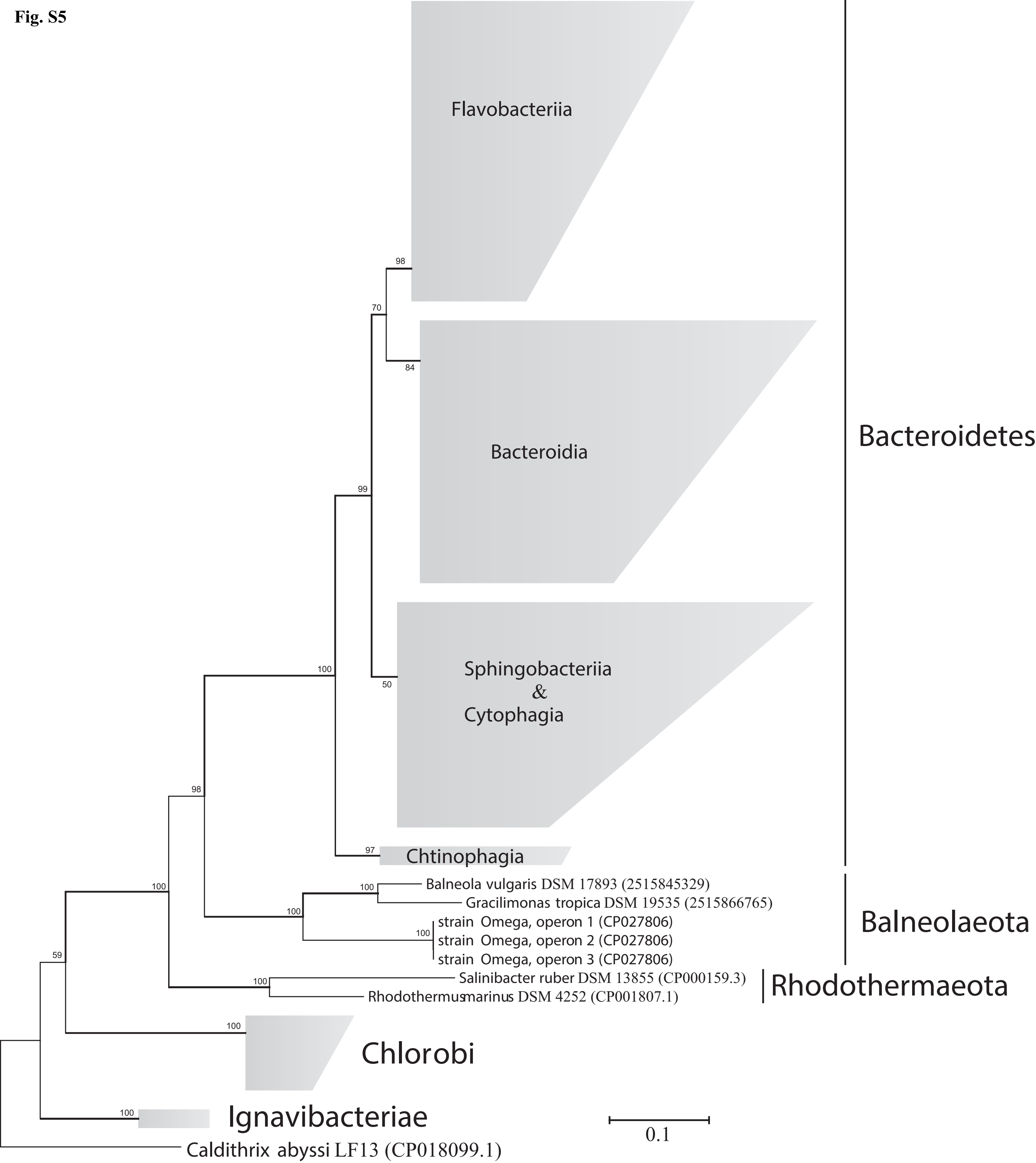
Flavobacteriia
98
70
84
Bacteroidia
99
100
Sphingobacteriia
&
Cytophagia
50
98
97
Chtinophagia
Bacteroidetes
100
Balneola vulgaris DSM 17893 (2515845329)
Gracilimonas tropica DSM 19535 (2515866765)
100
strain Omega, operon 1 (CP027806)
100
strain Omega, operon 2 (CP027806)
strain Omega, operon 3 (CP027806)
Balneolaeota
59
100
Salinibacter ruber DSM 13855 (CP000159.3)
Rhodothermus marinus DSM 4252 (CP001807.1)
Rhodothermaeota
100
Chlorobi
100
Ignavibacteriae
0.1
Caldithrix abyssi LF13 (CP018099.1)

Table S2

COG Function category	*Cyclonatronum proteivorans*		*Rhodothermus marinus* DSM 4252		*Salisaeta longa* DSM 21114		*Salinibacter ruber* M8		*Salinibacter ruber* M31		*Balneola vulgaris* DSM 17893		*Gracilimonas tropica* DSM 19535	
	hits	*share, %*	*hits*	*share, %*	*hits*	*share, %*	*hits*	*share, %*	*hits*	*share, %*	*hits*	*share, %*	*hits*	*share, %*
**Amino acid transport and metabolism**	171	8.56%	171	8.25%	135	7.25%	181	8.82%	183	9.34%	162	9.74%	175	8.22%
**Carbohydrate transport and metabolism**	99	4.96%	142	6.85%	98	5.27%	91	4.43%	92	4.69%	58	3.49%	102	4.79%
**Cell cycle control, cell division, chromosome partitioning**	27	1.35%	26	1.25%	26	1.40%	22	1.07%	20	1.02%	25	1.50%	28	1.31%
**Cell motility**	21	1.05%	52	2.51%	13	0.70%	72	3.51%	65	3.32%	10	0.60%	25	1.17%
**Cell wall/membrane/envelope biogenesis**	159	7.96%	144	6.95%	161	8.65%	150	7.31%	127	6.48%	130	7.82%	187	8.78%
**Chromatin structure and dynamics**	1	0.05%	2	0.10%	2	0.11%	2	0.10%	2	0.10%	1	0.06%	1	0.05%
**Coenzyme transport and metabolism**	108	5.41%	130	6.27%	117	6.29%	126	6.14%	126	6.43%	98	5.89%	123	5.77%
**Cytoskeleton**	0	0.00%	0	0.00%	1	0.05%	2	0.10%	1	0.05%	0	0.00%	1	0.05%
**Defense mechanisms**	82	4.11%	60	2.89%	49	2.63%	61	2.97%	56	2.86%	44	2.65%	71	3.33%
**Energy production and conversion**	120	6.01%	122	5.89%	114	6.13%	118	5.75%	118	6.02%	99	5.95%	130	6.10%
**Extracellular structures**	6	0.30%	5	0.24%	1	0.05%	5	0.24%	5	0.26%	5	0.30%	8	0.38%
**Function unknown**	86	4.31%	84	4.05%	95	5.10%	92	4.48%	77	3.93%	81	4.87%	111	5.21%
**General function prediction only**	209	10.47%	205	9.89%	176	9.46%	168	8.19%	162	8.27%	162	9.74%	201	9.44%
**Inorganic ion transport and metabolism**	95	4.76%	116	5.60%	120	6.45%	111	5.41%	113	5.77%	63	3.79%	109	5.12%
**Intracellular trafficking, secretion, and vesicular transport**	22	1.10%	27	1.30%	20	1.07%	27	1.32%	28	1.43%	24	1.44%	25	1.17%
**Lipid transport and metabolism**	90	4.51%	93	4.49%	99	5.32%	84	4.09%	81	4.13%	83	4.99%	96	4.51%
**Mobilome: prophages, transposons**	31	1.55%	12	0.58%	9	0.48%	39	1.90%	22	1.12%	3	0.18%	24	1.13%
**Nucleotide transport and metabolism**	68	3.41%	70	3.38%	64	3.44%	73	3.56%	72	3.67%	62	3.73%	65	3.05%
**Posttranslational modification, protein turnover, chaperones**	103	5.16%	105	5.07%	96	5.16%	100	4.87%	96	4.90%	91	5.47%	114	5.35%
**RNA processing and modification**	0	0.00%	1	0.05%	0	0.00%	0	0.00%	0	0.00%	0	0.00%	0	0.00%
**Replication, recombination and repair**	86	4.31%	73	3.52%	77	4.14%	86	4.19%	86	4.39%	80	4.81%	97	4.55%
**Secondary metabolites biosynthesis, transport and catabolism**	45	2.25%	55	2.65%	52	2.79%	57	2.78%	49	2.50%	54	3.25%	55	2.58%
**Signal transduction mechanisms**	108	5.41%	98	4.73%	90	4.84%	124	6.04%	119	6.07%	77	4.63%	101	4.74%
**Transcription**	89	4.46%	108	5.21%	84	4.51%	98	4.78%	97	4.95%	89	5.35%	109	5.12%
**Translation, ribosomal structure and biogenesis**	171	8.56%	172	8.30%	162	8.70%	163	7.94%	163	8.32%	162	9.74%	172	8.08%
**Not in COG**	1501	44.95%	1091	36.80%	1138	40.17%	1451	43.94%	1148	39.60%	990	39.27%	1495	43.64%

Fig. S6

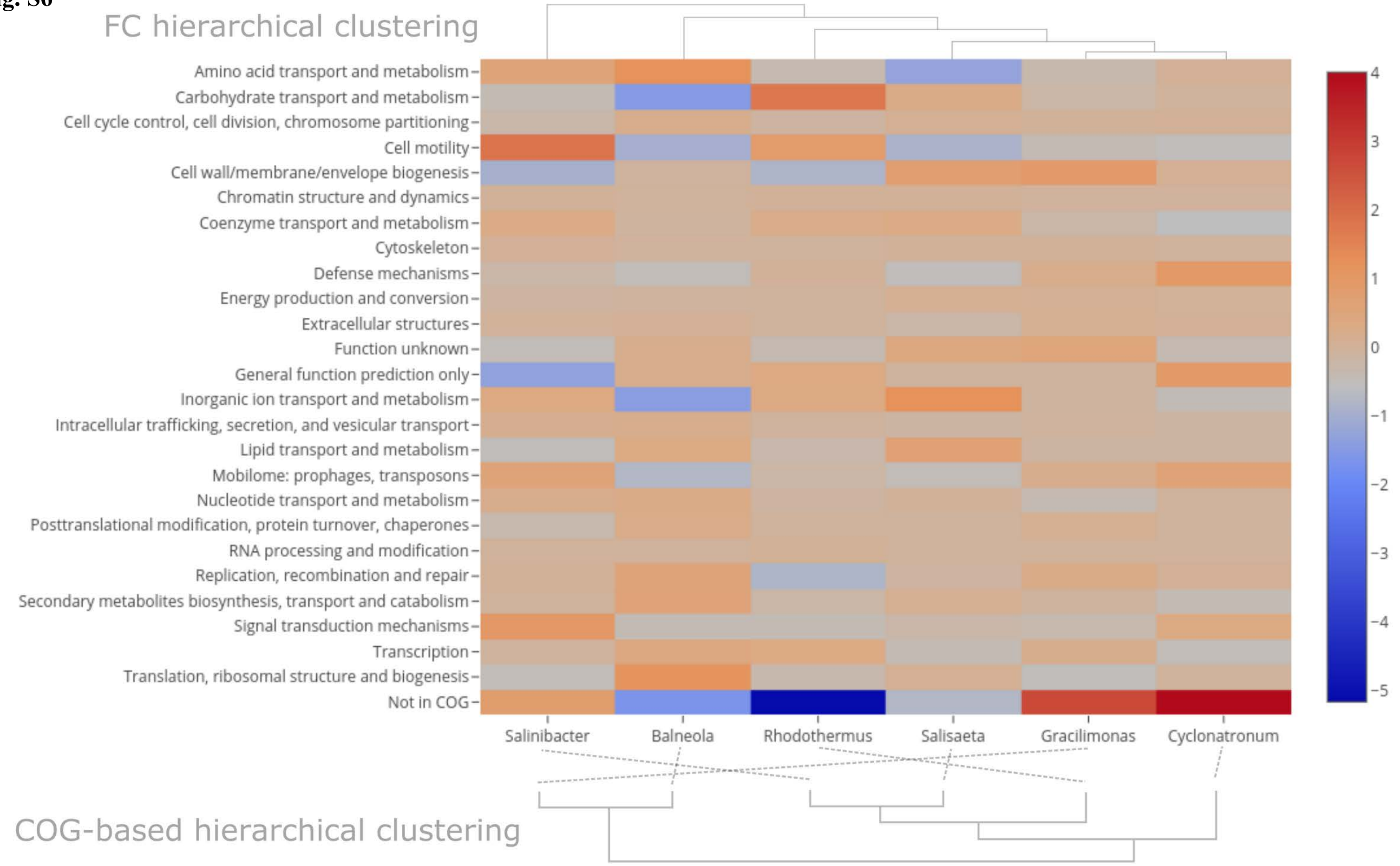
FC hierarchical clustering
Amino acid transport and metabolism
Carbohydrate transport and metabolism
Cell cycle control, cell division, chromosome partitioning
Cell motility
Cell wall/membrane/envelope biogenesis
Chromatin structure and dynamics
Coenzyme transport and metabolism
Cytoskeleton
Defense mechanisms
Energy production and conversion
Extracellular structures
Function unknown
General function prediction only
Inorganic ion transport and metabolism
Intracellular trafficking, secretion, and vesicular transport
Lipid transport and metabolism
Mobilome: prophages, transposons
Nucleotide transport and metabolism
Posttranslational modification, protein turnover, chaperones
RNA processing and modification
Replication, recombination and repair
Secondary metabolites biosynthesis, transport and catabolism
Signal transduction mechanisms
Transcription
Translation, ribosomal structure and biogenesis
Not in COG
Salinibacter
Balneola
Rhodothermus
Salisaeta
Gracilimonas
Cyclonatronum
4
3
2
1
0
−1
−2
−3
−4
−5
COG-based hierarchical clustering

Fig. S7

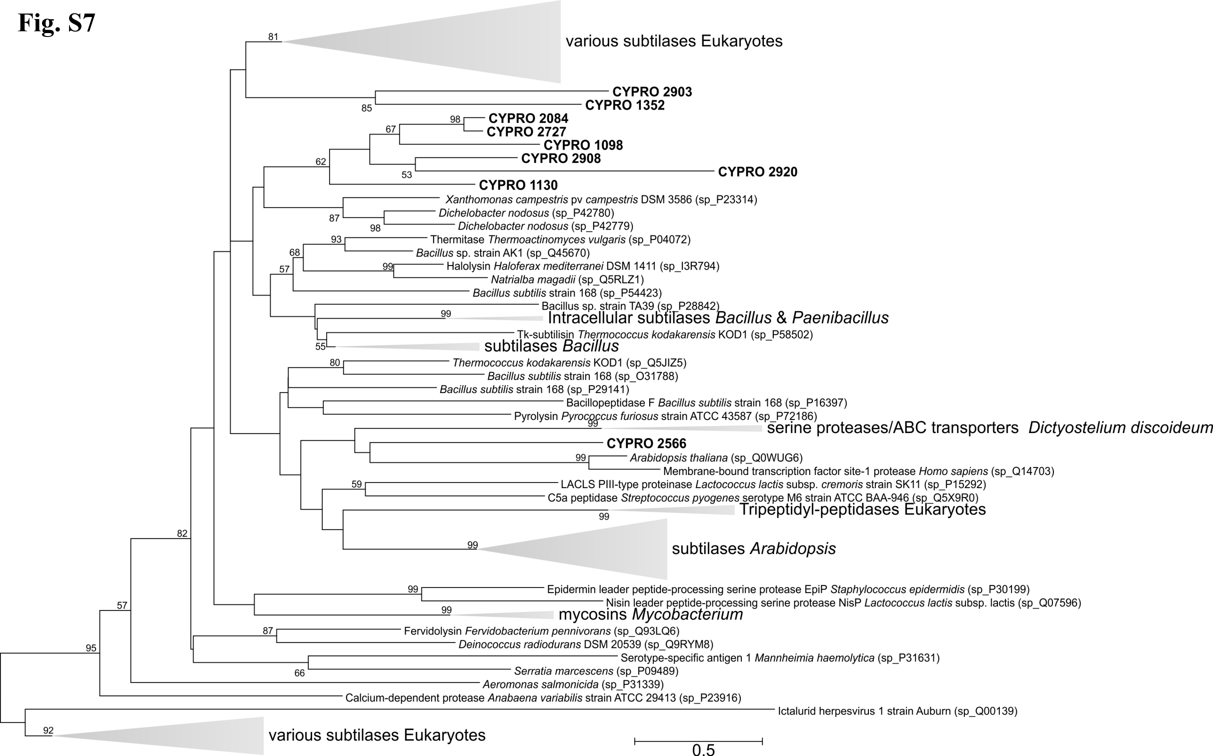
various subtilases Eukaryotes
81
CYPRO 2903
CYPRO 1352
85
CYPRO 2084
CYPRO 2727
98
67
CYPRO 1098
CYPRO 2908
CYPRO 2920
62
53
CYPRO 1130
Xanthomonas campestris pv campestris DSM 3586 (sp_P23314)
Dichelobacter nodosus (sp_P42780)
Dichelobacter nodosus (sp_P42779)
87
98
Thermitase Thermoactinomyces vulgaris (sp_P04072)
93
Bacillus sp. strain AK1 (sp_Q45670)
68
Halolysin Haloferax mediterranei DSM 1411 (sp_I3R794)
99
Natrialba magadii (sp_Q5RLZ1)
57
Bacillus subtilis strain 168 (sp_P54423)
Bacillus sp. strain TA39 (sp_P28842)
Intracellular subtilases Bacillus & Paenibacillus
99
Tk-subtilisin Thermococcus kodakarensis KOD1 (sp_P58502)
subtilases Bacillus
55
Thermococcus kodakarensis KOD1 (sp_Q5JIZ5)
80
Bacillus subtilis strain 168 (sp_O31788)
Bacillus subtilis strain 168 (sp_P29141)
Bacillopeptidase F Bacillus subtilis strain 168 (sp_P16397)
Pyrolysin Pyrococcus furiosus strain ATCC 43587 (sp_P72186)
serine proteases/ABC transporters Dictyostelium discoideum
99
CYPRO 2566
Arabidopsis thaliana (sp_Q0WUG6)
99
Membrane-bound transcription factor site-1 protease Homo sapiens (sp_Q14703)
LACLS PIII-type proteinase Lactococcus lactis subsp. cremoris strain SK11 (sp_P15292)
59
C5a peptidase Streptococcus pyogenes serotype M6 strain ATCC BAA-946 (sp_Q5X9R0)
Tripeptidyl-peptidases Eukaryotes
99
subtilases Arabidopsis
99
82
Epidermin leader peptide-processing serine protease EpiP Staphylococcus epidermidis (sp_P30199)
99
Nisin leader peptide-processing serine protease NisP Lactococcus lactis subsp. lactis (sp_Q07596)
mycosins Mycobacterium
99
57
Fervidolysin Fervidobacterium pennivorans (sp_Q93LQ6)
87
Deinococcus radiodurans DSM 20539 (sp_Q9RYM8)
95
Serotype-specific antigen 1 Mannheimia haemolytica (sp_P31631)
Serratia marcescens (sp_P09489)
66
Aeromonas salmonicida (sp_P31339)
Calcium-dependent protease Anabaena variabilis strain ATCC 29413 (sp_P23916)
Ictalurid herpesvirus 1 strain Auburn (sp_Q00139)
92
various subtilases Eukaryotes
0.5

Fig. S8

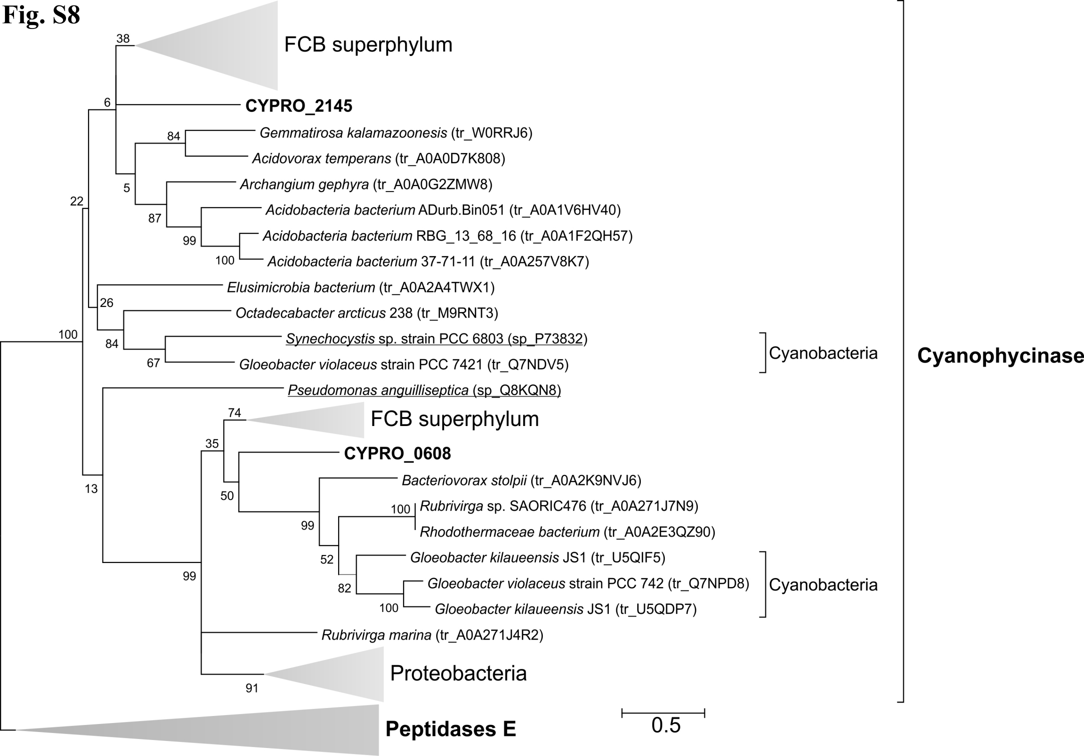

FCB superphylum
CYPRO_2145
Gemmatirosa kalamazoonesis (tr_W0RRJ6)
Acidovorax temperans (tr_A0A0D7K808)
Archangium gephyra (tr_A0A0G2ZMW8)
Acidobacteria bacterium ADurb.Bin051 (tr_A0A1V6HV40)
Acidobacteria bacterium RBG_13_68_16 (tr_A0A1F2QH57)
Acidobacteria bacterium 37-71-11 (tr_A0A257V8K7)
Elusimicrobia bacterium (tr_A0A2A4TWX1)
Octadecabacter arcticus 238 (tr_M9RNT3)
Synechocystis sp. strain PCC 6803 (sp_P73832)
Gloeobacter violaceus strain PCC 7421 (tr_Q7NDV5)
Cyanobacteria
Pseudomonas anguilliseptica (sp_Q8KQN8)
FCB superphylum
CYPRO_0608
Bacteriovorax stolpii (tr_A0A2K9NVJ6)
Rubrivirga sp. SAORIC476 (tr_A0A271J7N9)
Rhodothermaceae bacterium (tr_A0A2E3QZ90)
Gloeobacter kilaueensis JS1 (tr_U5QIF5)
Gloeobacter violaceus strain PCC 742 (tr_Q7NPD8)
Gloeobacter kilaueensis JS1 (tr_U5QDP7)
Cyanobacteria
Rubrivirga marina (tr_A0A271J4R2)
Proteobacteria
Peptidases E
Cyanophycinase
0.5
38
6
84
5
22
87
99
100
26
100
84
67
74
35
13
50
99
100
52
82
100
99
91
